# Metabolomic and microbiomic insights into color changes during the sweating process in *Dipsacus asper*

**DOI:** 10.3389/fmicb.2023.1195088

**Published:** 2023-08-29

**Authors:** Hua He, Jiao Xu, Taimin Zhou, Yang Yang, Changgui Yang, Chenghong Xiao, Chenggang Zhang, Liangyuan Li, Tao Zhou

**Affiliations:** Resource Institute for Chinese and Ethnic Materia Medica, Guizhou University of Traditional Chinese Medicine, Guiyang, China

**Keywords:** *Dipsacus asper*, metabolomics, microbiome, sweating, color changes

## Abstract

Sweating is one of the most important primary processing methods of Chinese medicinal materials. *Dipsacus asper* is a typical representative of sweating treatment that is recommended by the Chinese Pharmacopoeia. The color change of the fracture surface of the root is the prominent feature of sweating treatment. However, few studies have focused on the mechanism of color change during sweating treatment. In this study, widely targeted metabolomics and ITS high-throughput sequencing technologies were applied to detect metabolites and microbial structure and diversity in the root of *D. asper* during sweating treatment. A total of 667 metabolites, including 36 downregulated and 78 upregulated metabolites, were identified in *D. asper* following sweating treatment. The significantly differential metabolites were divided into 12 classes, including terpenoids and phenolic acids. Moreover, all the differential terpenoids were upregulated and 20 phenolic acids showed a significant change after sweating treatment. In addition, microbial community diversity and richness increased following sweating treatment. The composition of microbial communities revealed that the relative abundances of Ascomycota and Basidiomycota significantly changed after sweating treatment. Correlation analysis revealed that Ascomycota (*Fusarium* sp., *Macrophomina* sp., *Ilyonectria* sp., *Memnoniella* sp., *Penicillium* sp., *Cyphellophora* sp., *Neocosmospora* sp., unclassified_f_Nectriaceae, and unclassified_o_Saccharomycetales) and Basidiomycota (*Armillaria* sp.) were associated with the content of terpenoids (6-deoxycatalpol and laciniatoside III) and phenolic acids (3-(4-hydroxyphenyl)-propionic acid, ethyl caffeate, 4-O-glucosyl-4-hydroxybenzoic acid, 2-acetyl-3-hydroxyphenyl−1-O-glucoside, 4-O-glucosyl-3,4-dihydroxybenzyl alcohol, 3-O-feruloylquinic acid, 3,4-O-dicaffeoylquinic acid methyl ester, O-anisic acid, and coniferyl alcohol). We speculate that the Ascomycota and Basidiomycota affect the content of terpenoids and phenolic acids, resulting in color change during sweating treatment in *D. asper.* This study provides a foundation for analyzing the mechanism involved in the processing of Chinese medicinal materials.

## Introduction

Chinese medicinal herbs have played a vital role in clinical therapy for thousands of years, and most of them should carry out primary processing to form clinical medicinal materials. The primary processing of Chinese medicinal materials includes cleaning, steaming, boiling, sweating, and drying, and the different processing methods can affect the characteristics and quality of the materials (Wu et al., 2019). Sweating is a special processing method in which fresh herbs are harvested and semi-dried before being piled up and heated, resulting in the internal water of the fresh herbs evaporating outward and appearing on the surface of the herbs; this is known as sweating or “diaphoretic” processing in the Chinese Pharmacopoeia (version 2020; Tao et al., 2020). It has been reported that sweating can accelerate the drying rate of medicinal materials by redistributing the internal water, and increase their flavor or reduce their irritation (Duan et al., 2013). Furthermore, sweating affects the temperature and humidity inside the medicinal materials, which then changes the microbial communities and the activity of enzymes in biological tissues. These changes may provide conditions for the biological and chemical conversion of primary or secondary metabolites, directly influencing the quality of medicinal materials (Chen et al., 2018; Wu et al., 2019).

Five Chinese medicinal materials have been clearly proposed to require sweating, including *Eucommia ulmoides* Oliv., *Magnolia officinalis* Rehd. et Wils, and *Dipsacus asper* Wall. ex C.B. Clarke. Color change is an important characteristic during sweating treatment. Study of *M. officinalis* has suggested that sweating can affect the contents of the main active substances, and this change may be caused by the variation in the microbial community during the sweating process (Wu et al., 2019). Moreover, previous studies have shown that the change in the appearance and color of medicinal materials is due to a series of physicochemical reactions that cause the internal chemical composition change (Zhu et al., 2022). The color change during sweating may be related to the change in the metabolites or the variation in the microbial community. However, the mechanism between color change and sweating in Chinese medicine has not yet been explained.

**Figure 1:**
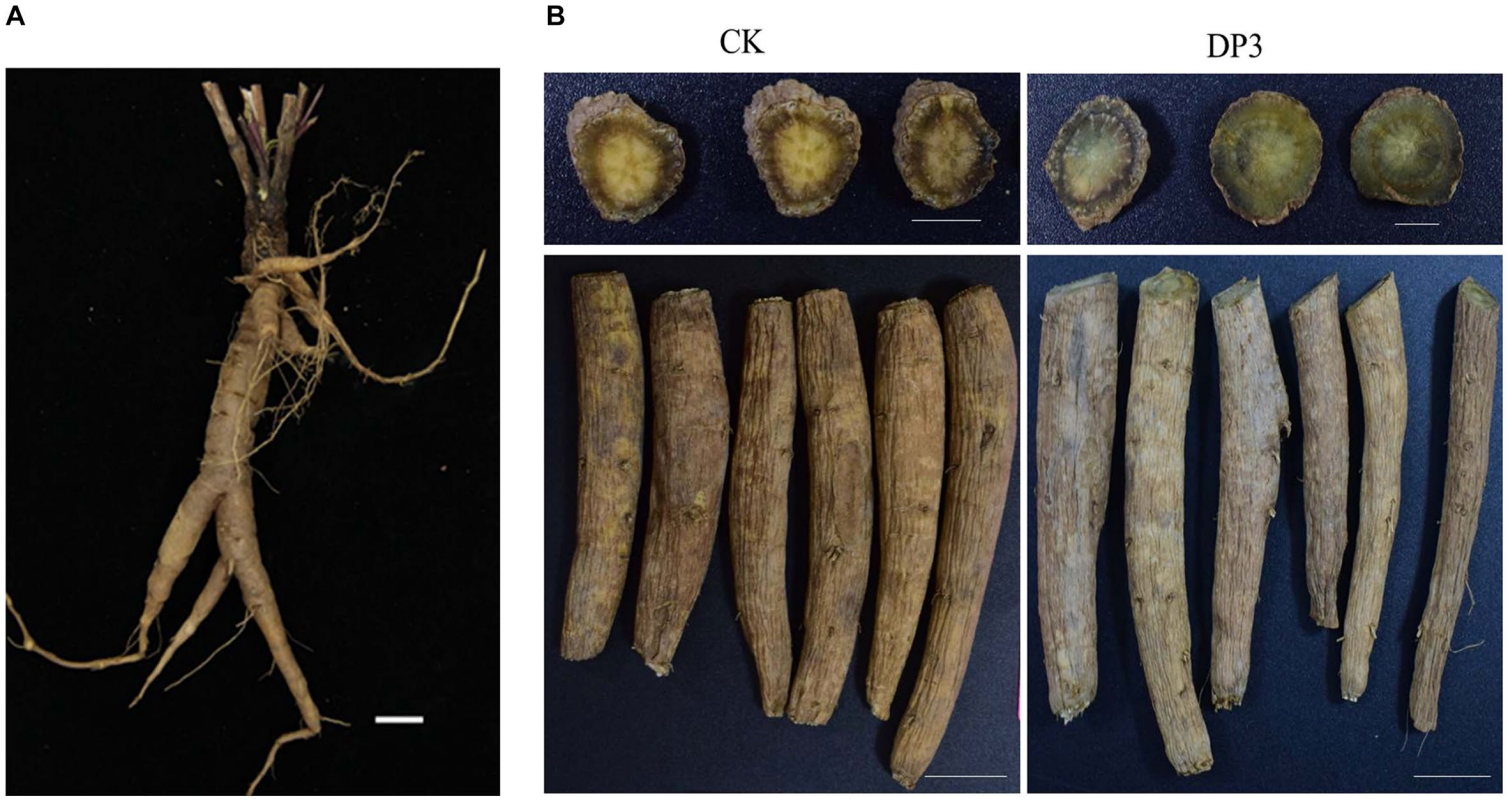
Phenotype of the root in *Dipsacus asper.* **(A)** The fresh root. **(B)** The root before (CK) and after (DP3) sweating. Scale bars = 2 cm.

*D. asper* is an important traditional Chinese herb. The root is a Chinese traditional medicine that undergoes sweating treatment, and it is a well-known medicine used for the treatment of bone diseases, traumatic hematoma, and uterine bleeding (Niu et al., 2011; Yu et al., 2012a,b; Tao et al., 2020). Pharmacological research has revealed that *D. asper* plays an important role in the treatment of depression (Zhang et al., 2020). Previous studies have isolated approximately 100 components from *D. asper*, including triterpenoids, iridoids, phenolic acids, essential oils, alkaloids, lignin, and fatty acids, and triterpenoid saponins are the major bioactive compound (Jung et al., 2012). A study investigating the spectrum-effect relationship between sweated and crude *D. asper* in terms of cell proliferation and differentiation suggested that sweating can affect the efficacy of *D. asper* by changing the content of its main material basis, including asperosaponin VI, loganin, and caffeic acid (Yang et al., 2019). In addition, sweating can change the color of the fracture surface of *D. asper* medicine (Figure 1), which is regarded as one of the important indicators of quality in *D. asper*. However, it is not clear what causes the color change and how the color change relates to the variation of compounds and microorganisms in this valuable Chinese herbal medicine.

Our previous study also showed that sweating can affect the content of total triterpene saponins and asperosaponin VI, which is the index component of *D. asper* (He et al., 2021), while the content of asperosaponin VI in *D. asper* showed no correlation with the color change of the cross-section in stable sweating conditions (Zhou et al., 2021). These results implied that color change during the sweating process may be related to other metabolites. Based on the above results, widely targeted metabolomics was used to comprehensively analyze metabolite composition and relative content in *D. asper* root between the control and the sweating group. Then, high-throughput sequencing technology was used to analyze fungal community variations between the two groups. Metabolic and microbiome association analysis was conducted to find the metabolites and fungi that led to the color change following sweating treatment of *D. asper* root. These results would provide insight into the very nature of sweating processing in Chinese medicine.

## Materials and methods

### Plant materials and sample preparation

*D. asper* plants were cultivated for more than 2 years at Guizhou University of Traditional Chinese Medicine, Guizhou Province, China. The fresh root of *D. asper* was collected and washed. Then, the roots were cut into approximately 10-cm-thick sections. All the samples were divided in half for sweating treatment.

### Sweating treatment

Fresh *D. asper* was collected and processed according to the sweating method established in our previous study (Zhou et al., 2021). All the samples were dried in a dryer until the relative water content reached 40%, and these samples were used for sweating processing. One part of these samples was taken as the control, named CK in the subsequent analysis. The other samples were sweated for 3 days at 25°C in an airtight environment and were named DP3. These two samples were stored in a − 80°C freezer for widely targeted metabolomics and high-throughput sequencing, simultaneously. There were three biological replicates per treatment.

### Metabolite extraction

The above samples with three biological replicates were subjected to widely targeted metabolomic analysis (Metware Biotechnology Co., Ltd. Wuhan, China). The samples were freeze-dried with a vacuum freeze-dryer (Scientz-100F) and then crushed using a mixer mill (MM 400, Retsch) with a zirconia bead for 1.5 min at 30 Hz. The lyophilized powder (100 mg) was dissolved in 1.2 mL of 70% methanol solution, vortexed for 30 s every 30 min (for a total of six times), and then stored at 4°C overnight. Following centrifugation at 12,000 rpm for 10 min, the extracts were filtrated (0.22 μm pore size) before ultra-high-performance liquid chromatography–tandem mass spectrometry (UHPLC–MS/MS) analysis.

### UHPLC-ESI-Q TRAP-MS/MS and analysis

The sample extracts were analyzed using an UHPLC-ESI-MS/MS system (UHPLC, SHIMADZU Nexera X2, Shanghai, China; MS, Applied Biosystems 4,500 Q TRAP, Thermo Fisher) with an Agilent SB-C18 column (1.8 μm, 2.1 mm × 100 mm). The mobile phase consisted of solvent A (pure water with 0.1% formic acid) and solvent B (acetonitrile with 0.1% formic acid). The effluent was alternatively connected to an ESI-triple quadrupole-linear ion trap (QTRAP)-MS.LIT and triple quadrupole (QQQ) scans were acquired on a mass spectrometer (Q TRAP; AB4500 Q TRAP UHPLC/MS/MS System) equipped with an ESI Turbo Ion-Spray interface, operating in positive and negative ion mode and controlled by Analyst 1.6.3 software (AB Sciex). Based on the self-built database MWDB V2.0 (Metware Biotechnology Co., Ltd. Wuhan, China), primary and secondary mass spectrometry data were subjected to qualitative analysis. Isotopic signals, repeating signals containing K^+^ ions, Na^+^ ions, NH4^+^ ions, and fragment ions that were other larger molecular weight compounds were removed from the analysis. Moreover, the accurate mass, MS/MS spectra, and retention time (RT) of the identified metabolites in the CK and DP3 samples were also compared with the compounds in the self-built database MWDB V2.0 to improve the accuracy of the identification. The comparison was performed with the following parameters: the mass error of the parent ion and fragment ion of the metabolites was set below 20 ppm, and the RT values were set with a variation of less than 0.2 min. Metabolite quantification was performed using triple quadrupole mass spectrometry in multiple reaction monitoring (MRM) (Fraga et al., 2010).

Unsupervised principal component analysis (PCA) was performed using R software (www.r-project.org/). The clustering heat map was analyzed with normalized data. Significantly different accumulated metabolites (DAMs) between groups were determined by variable importance in projection (VIP) of ≥1 and absolute log2 (fold change) of ≥1.

### DNA extraction and its amplification

Microbial DNA was extracted from the two sample groups mentioned above (CK, DP3), each with three biological replicates, using an E.Z.N.A.® Soil DNA Kit (Omega Bio-tek, Norcross, GA, United States) according to the manufacturer’s protocols. Total DNA concentration and quality were determined using a NanoDrop 2000 UV–vis spectrophotometer (Thermo Scientific, Wilmington, United States) and agarose gel electrophoresis. The ITS regions of the fungal 18S rRNA genes were amplified with primers ITS1F (5′-CTTGGTCATTTAGAGGAAGTAA-3′) and ITS2R (5′-GCTGCGTTCTTCATCGATGC-3′). The PCR reactions were conducted using the following protocol: 95°C for 3 min, followed by 36 cycles at 95°C for 30 s, 55°C for 30 s, and 72°C for 45 s, with a final extension at 72°C for 10 min. The PCRs (Gene Amp 9,700, ABI, United States) were performed in triplicate. Amplicons were extracted from 2% agarose gels, further purified using an AxyPrep DNA Gel Extraction Kit (Axygen Biosciences, Union City, CA, United States), and quantified using QuantiFluor™-ST (Promega, United States) according to the manufacturer’s protocol.

### Illumina MiSeq sequencing

Purified amplicons were pooled in equimolar ratios and paired-end sequenced (2 × 300) on an Illumina MiSeq platform (Illumina, San Diego, United States) according to the standard protocols described by Majorbio Bio-Pharm Technology Co. Ltd. (Shanghai, China). After Illumina sequencing, the fungi raw sequences were deposited in the Sequence Read Archive database at the NCBI (the accession number: PRJNA1005339). Raw fastq files were demultiplexed, quality-filtered using Trimmomatic, and merged using FLASH with the following criteria: (i) the reads were truncated at any site receiving an average quality score of <20 over a 50 bp sliding window; (ii) primers were exactly matched, allowing two nucleotide mismatching, and reads containing ambiguous bases were removed; and (iii) sequences with overlaps longer than 10 bp were merged according to their overlap sequence.

### Sequencing data analysis

Operational taxonomic units (OTUs) were clustered with a 97% similarity cutoff using UPARSE (version 7.0 http://drive5.com/uparse/), and chimeric sequences were identified and removed using USEARCH. The taxonomy of each 18S rRNA gene sequence was analyzed using the RDP Classifier algorithm (version 2.2 http://sourceforge.net/projects/rdp-classifier/) against the Silva (SSU123) database using a confidence threshold of 70%.

Alpha diversity indices were used to evaluate microbial abundance and diversity. Chao, ACE indexes, and principal coordinate analysis (PCoA) were analyzed using the online tool of the Majorbio Cloud Platform (Ren et al., 2022). Unweighted Pair-group Method with Arithmetic Mean (UPGMA) was used to construct a phylogenetic tree to investigate beta-diversity patterns.

### Correlation analysis

Correlation analysis was performed on the differential fungi and metabolites detected in each group. The Spearman correlation coefficient of microorganisms and metabolites was calculated (Franzosa et al., 2019). The Spearman correlation value *r* is between −1 and + 1. Green indicates a positive correlation with *r* > 0, and red represented a negative correlation with *r* < 0. And *p* - value below 0.05 indicates a significant correlation. Metabolites with a correlation greater than 0.8 and a correlation significance test *p* - value <0.05 were used to generate the chord diagram and heatmap.

## Results and discussion

### Phenotype observation after sweating in *Dipsacus asper*

Sweating treatment is a traditional processing method in *D. asper*. According to the Chinese Pharmacopoeia and a previous study (Zhou et al., 2021), the roots of *D. asper* were used for sweating treatment (Figure 1A). After sweating treatment, the color of the cross-section in DP3 changed from yellow to green, which was consistent with the Chinese Pharmacopoeia description (version 2020) (Figure 1B). There was little difference in the root epidermis and root morphology, and color change was the key feature of sweating treatment. It has been shown that the contents of protodioscin, diosgenin, and narcissoside have a significantly positive correlation with color change during the processing of *Polygonatum kingianum* Rhizoma (Wang et al., 2022). Previous studies have indicated that carotenoids play an important role in the colors that mostly range from yellow to red, which affected the coloration in plants (Nisar et al., 2015; Zheng et al., 2019). The accumulation of anthocyanins in some mature fruits, including grapes, strawberries, and apples, contributed to fruits with rich colors (Zhang et al., 2008). Moreover, the content of tanshinone and salvianolic acid is involved in the root color of *Salvia miltiorrhiza* (Deng et al., 2020; Hao et al., 2020). In conclusion, metabolites such as carotenoids, anthocyanins, saponins, and phenolic acids were related to color changes. Hence, we speculated that metabolite changes led to color changes after sweating treatment in *D. asper*.

### Metabolites in control and sweated *Dipsacus asper*

**Figure 2:**
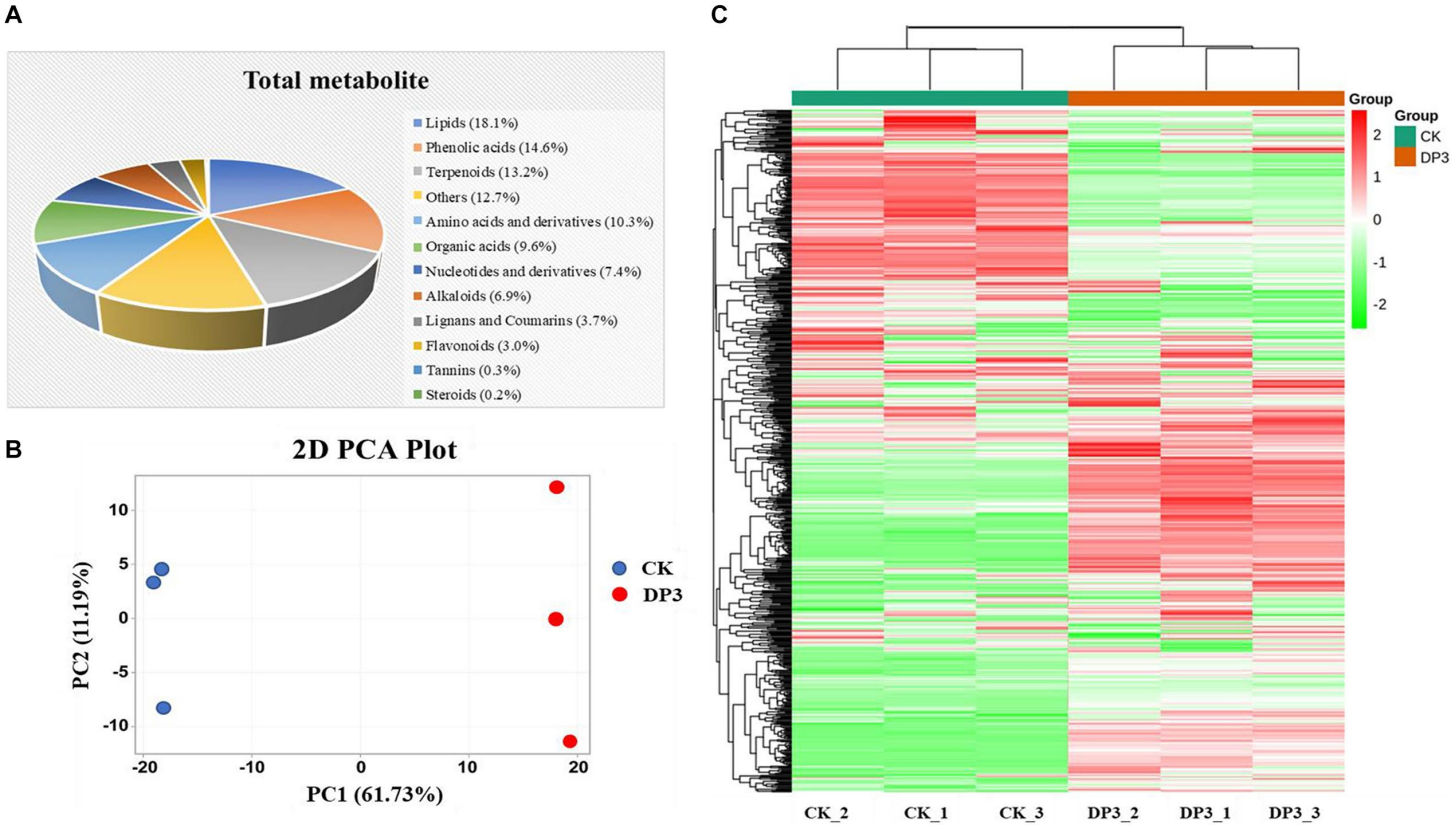
Metabolites detected in *D. asper*. **(A)** Classification and proportion of 667 metabolites detected in *D. asper*. **(B)** Principal component analysis (PCA) of *D. asper* samples. **(C)** Hierarchical clustering heatmap of metabolite accumulation in *D. asper*.

To investigate the relationship between metabolites and sweating treatment, widely targeted metabolomic analysis was performed with control (CK) and sweated (DP3) *D. asper*. A total of 667 metabolites were identified. The metabolites could be classified into 12 categories: including terpenoids, tannins, phenolic acids, organic acids, nucleotides and derivatives, lipids, lignans and coumarins, flavonoids, steroids, amino acid and derivatives, alkaloids, and other metabolites (Figure 2A). The detailed information of these metabolites is shown in Supplementary Table 1. Among them, the classifications with the highest number of metabolites were lipids (18.1%), phenolic acids (14.6%), and terpenoids (13.2%). These three types of metabolites were considered the main components in *D. asper.* Previous studies have shown that there are 52 common components in raw and sweated *D. asper* based on UPLC-Triple-TOF/MS analysis, including triterpenoid saponins, iridoids, and phenolic acids (Hong et al., 2020). We also identified a variety of metabolites in *D. asper* with sweating treatment, and there was a large number of alkaloids and lipids in addition to terpenoids and phenolic acids.

Moreover, PCA analysis showed that these two samples were clearly distinguishable, with PC1 and PC2 accounting for 61.73% and 11.19% of the variance, respectively (Figure 2B). There was a significant difference in PC1 between CK and DP3. The results indicated that the metabolite compositions of CK and DP3 were different. Moreover, the hierarchical clustering heatmap also showed that the metabolites were quite different following sweating treatment (Figure 2C). The results revealed that the differential metabolites may be responsible for color changes caused by sweating treatments in *D. asper*.

### Differential metabolites in control and sweated *Dipsacus asper*

**Figure 3:**
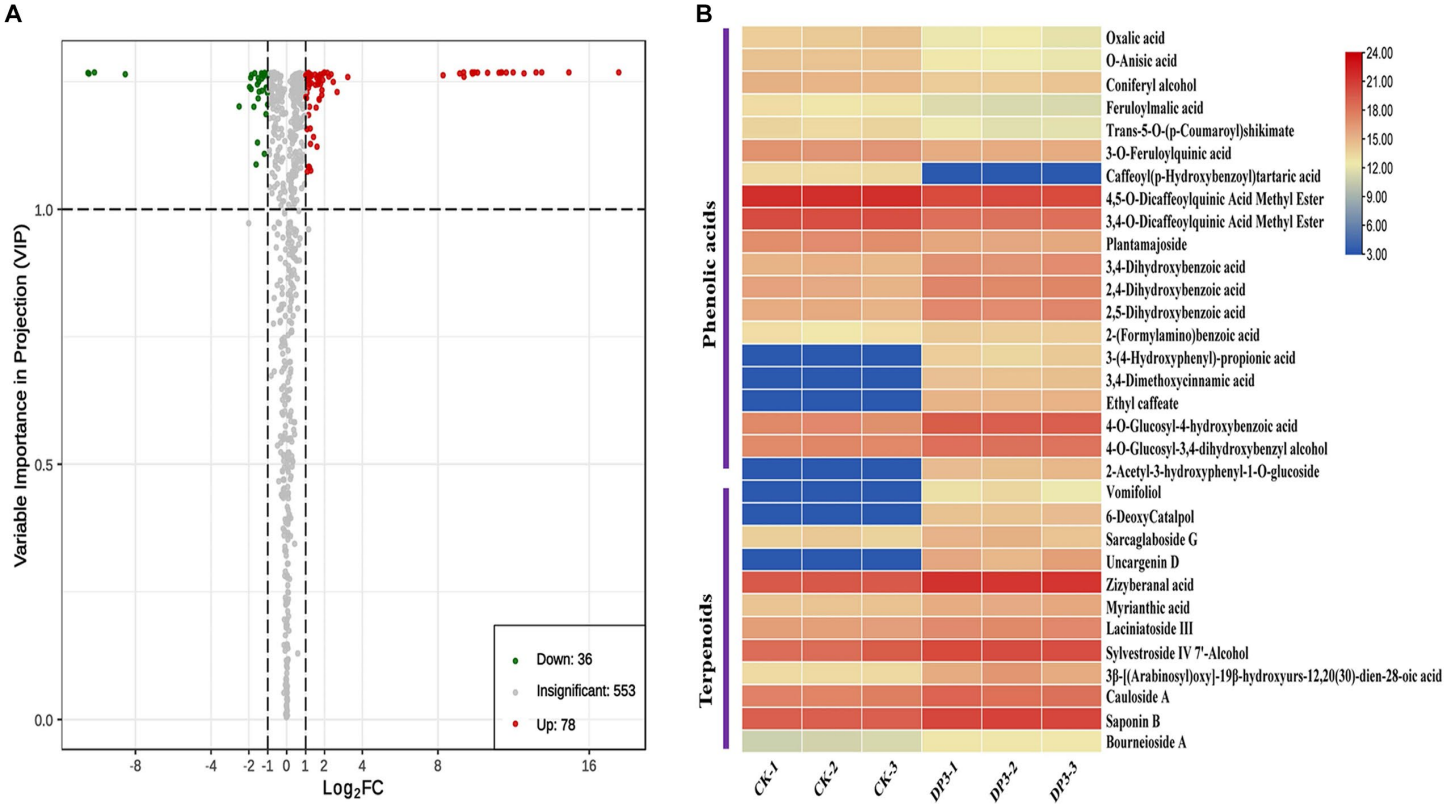
Differential metabolites analysis. **(A)** Volcano plots of differential metabolites between CK and DP3. **(B)** Cluster analysis of differential metabolites between CK and DP3.

To better understand the effects of the sweating treatment on the changes of the metabolites, a comparison of metabolites between control and sweated *D. asper* was analyzed. As shown in the volcano plot, out of the 667 metabolites detected, there were 114 significantly different accumulated metabolites after sweating, including 36 downregulated and 78 upregulated metabolites (Figure 3A; Supplementary Figure S1). Furthermore, the heatmap showed that the metabolites from all the 12 classes significantly differed between control and sweated *D. asper*. Based on the number and proportion of the significantly different accumulated metabolites, sweating mainly affected the content of the metabolites from the classes of phenolic acids, organic acids, amino acids and derivatives, and terpenoids (Supplementary Figure S2).

There were more phenolic acids and terpenoids among the significantly differential compounds (Supplementary Figure S3). All the terpenoids were upregulated and showed a significant change after sweating treatment, including two iridoids, laciniatoside III, and 6-deoxycatalpol (Figure 3B). Iridoids represent a large group of cyclopentano[*c*]pyran monoterpenoids, which are widely distributed in medicinal plants (Zhao and Shi, 2011). Owing to the unstable nature of their C_1_-OH group, iridoids often react with sugar to form glycosides, and they often decompose and transform during the processing of traditional Chinese medicines. Based on the structure and properties of iridoids, factors such as temperature, relative humidity, pH, and water content can affect the decomposition and transformation of iridoids during processing (Wang et al., 2020; Du et al., 2021). According to a previous study and Chinese Pharmacopoeia, *Gentiana macrophylla*, a Chinese medicinal herb, also needs sweating. It has been shown that the color change is related to secoiridoid glycoside content during the sweating processing (Wang et al., 2017). In our study, laciniatoside III is a bis-iridoid that was upregulated after the sweating treatment, and was first isolated from the aerial parts of *D. laciniatus* (Kocsis and Szabó, 1993). The iridoid glycoside 6-deoxycatalpol has a close relationship with catalpol, which also significantly correlates with the apparent color during the processing of Rehmanniae Radix (Chen et al., 2022). Hence, we think that the two iridoids, laciniatoside III and 6-deoxycatalpol, might play an important role in the color change of *D. asper* sweating.

In addition, 20 phenolic acids showed a significant content change compared with the control (Table 1). The phenolic acid metabolites, such as oxalic acid, O-anisic acid, and caffeoyl tartaric acid, were the downregulated metabolites with large fold changes after sweating treatment, whereas metabolites such as ethyl caffeate, 2-acetyl-3-hydroxyphenyl−1-O-glucoside, and 3,4-dimethoxycinnamic acid were the upregulated metabolites with large fold changes after sweating treatment (Figure 3B; Supplementary Figure S3).

**Table 1:** The differential phenolic acid compounds in control and sweated *Dipsacus asper*.

Compounds	Standard	VIP	Fold_Change	Log_2_FC	Type
Oxalic acid	Standard	1.24E+00	2.72E-01	−1.88E+00	Down
O-Anisic acid (2-methoxybenzoic acid)	Standard	1.26E+00	2.68E-01	−1.90E+00	Down
3,4-Dihydroxybenzoic acid (protocatechuic acid)*	Standard	1.24E+00	3.12E+00	1.64E+00	Up
2,4-Dihydroxybenzoic acid*	Standard	1.22E+00	3.29E+00	1.72E+00	Up
2,5-Dihydroxybenzoic acid; gentisic acid*	Standard	1.25E+00	3.46E+00	1.79E+00	Up
2-(Formylamino)benzoic acid	Spectrum	1.22E+00	2.07E+00	1.05E+00	Up
3-(4-Hydroxyphenyl)-propionic acid	Standard	1.27E+00	1.58E+03	1.06E+01	Up
Coniferyl alcohol	Standard	1.21E+00	4.97E-01	−1.01E+00	Down
3,4-Dimethoxycinnamic acid	Spectrum	1.27E+00	2.65E+03	1.14E+01	Up
Ethyl caffeate	Standard	1.27E+00	4.03E+03	1.20E+01	Up
4-O-Glucosyl-4-hydroxybenzoic acid	Spectrum	1.26E+00	4.68E+00	2.23E+00	Up
4-O-Glucosyl-3,4-dihydroxybenzyl alcohol	Spectrum	1.25E+00	2.19E+00	1.13E+00	Up
Feruloylmalic acid	Spectrum	1.25E+00	3.60E-01	−1.47E+00	Down
2-Acetyl-3-hydroxyphenyl-1-O-glucoside	Spectrum	1.27E+00	3.13E+03	1.16E+01	Up
Trans-5-O-(p-Coumaroyl)shikimate	Spectrum	1.23E+00	3.64E-01	-1.46E+00	Down
3-O-Feruloylquinic acid	Spectrum	1.27E+00	4.46E-01	-1.16E+00	Down
Caffeoyl(p-Hydroxybenzoyl)tartaric acid	Spectrum	1.27E+00	8.68E-04	-1.02E+01	Down
4,5-O-Dicaffeoylquinic acid methyl ester	Spectrum	1.27E+00	3.96E-01	-1.34E+00	Down
3,4-O-Dicaffeoylquinic acid methyl ester	Spectrum	1.27E+00	3.11E-01	-1.69E+00	Down
Plantamajoside	Standard	1.26E+00	4.12E-01	-1.28E+00	Down

Phenolic acids, metabolites that contain phenolic hydroxyl and carboxyl groups, exist in many medicine plants. In plant tissues, most of the phenolic acids are bound phenolics and occur in the form of esters and insoluble bound complexes. In addition to their important pharmacological activities, they are involved in the color change in Chinese medicinal herbs. Yang reported that the color change in *Lycii Fructus* was strongly correlated with a water-soluble pigment extract solution, which mainly contains phenolic acids and rutin (Yang et al., 2015). Additionally, phenols are involved in the color change in the primary processing of the roots of *Rehmannia glutinosa* with the action of the polyphenol oxidase (Duan et al., 2013). A study on the marked greening observed in some foods during food processing suggested that the reaction of chlorogenic acid or caffeic acid ester with a primary amino compound can form a green pigment compound (Yabuta et al., 2001). In our study, ethyl caffeate was upregulated after sweating, and amino acids were also present in the sweating samples; therefore, we speculated that ethyl caffeate may be involved in the green color formation in sweated *D. asper.*

Hence, all these results showed that terpenoids and phenolic acids play a pivotal role in the green color formation following sweating treatment. Factors leading to changes in metabolites that cause the cross-section color change during sweating would be the focus of our next study.

Given that sweating can affect the structure of microbial communities and the activity of enzymes in the tissues, and microbial communities can influence the transformation of the metabolites (Zhang et al., 2019), we speculated that the microbial community variation between the CK and DP3 groups could mediate the changes in differential metabolites.

### Diversity and composition analysis of fungal communities in control and sweated Dipsacus asper

**Figure 4:**
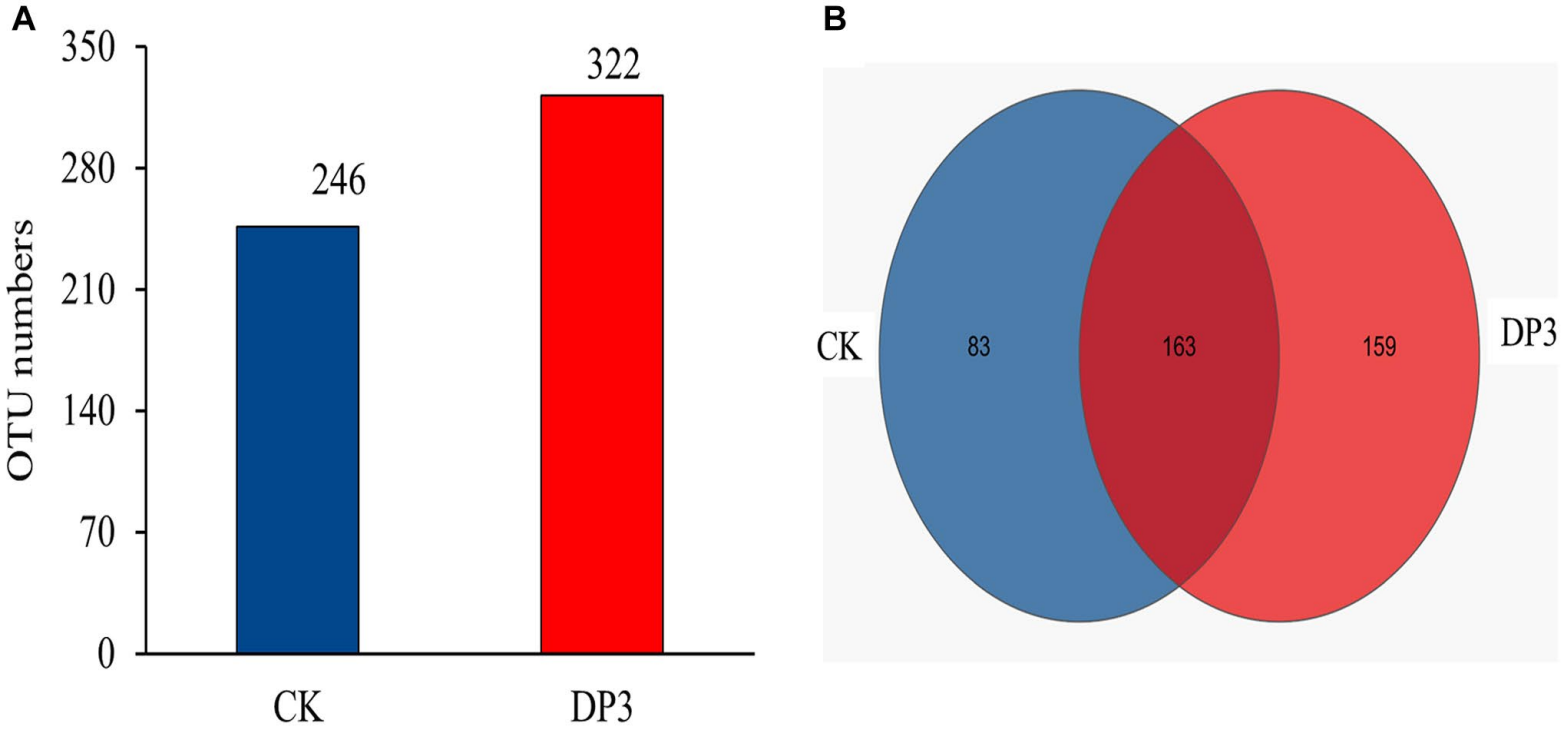
OTU numbers and Venn diagram analysis. **(A)** Number of OTUs in the fungal community. **(B)** Venn diagram of the fungal community.

To find out the differential microbial communities in control and sweated *D. asper*, high-throughput sequencing of the above two samples was completed. A total of 314,688 quality-filtered 18S rRNA gene sequences were obtained with an average length of 255 bp. The coverage index values of these two samples exceeded 99%. This suggested that most of the fungal communities could be detected and that the results could be used for further analysis. In our study, 246 and 322 OTUs were obtained for the fungal communities in CK and DP3, respectively, based on 97% similarity (Figure 4A). A total of 163 OTUs were the same in CK and DP3 (Figure 4B).

**Figure 5:**
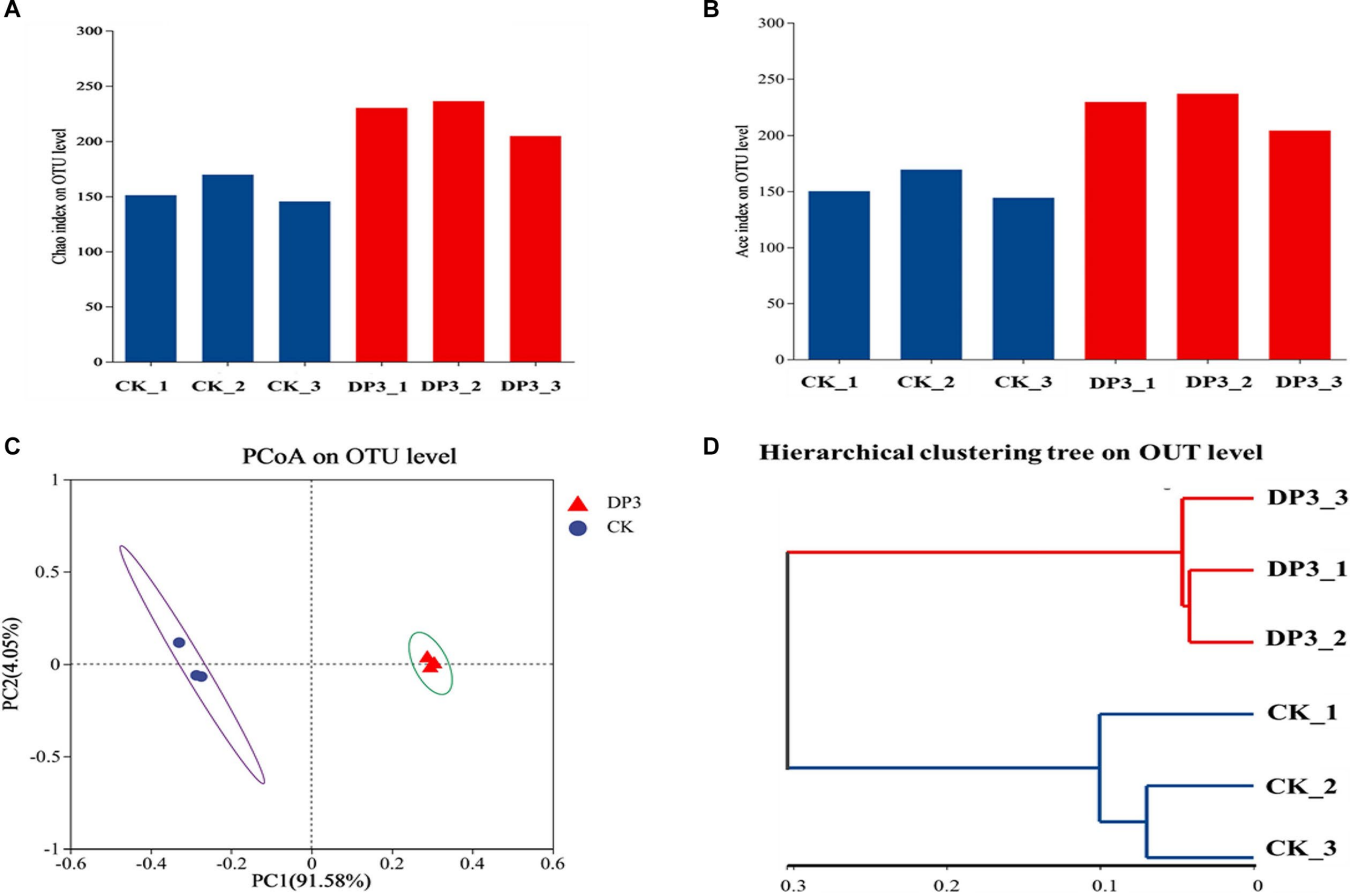
Fungal diversity analysis. The Chao indices **(A)** and ACE indices **(B)** of the fungal community. **(C)** PCoA analysis of the fungal community. **(D)** Hierarchical clustering analysis of the fungal community.

To investigate the microbial diversity and richness of the microbial communities in CK and DP3, alpha-diversity analysis was conducted. The Chao index based on ITS data showed that DP3 had a higher diversity and species richness than CK (Figure 5A). The Ace index showed similar results (Figure 5B). Hence, the results revealed that sweating treatment could elevate the richness and diversity in the root of *D. asper*. The alteration patterns of the microbial communities in CK and DP3 were investigated through beta diversity. Principal coordinate analysis (PCoA) showed that the microbial communities were separated between DP3 and CK by sweating treatment (Figure 5C), and the result of hierarchical clustering analysis was similar to that of PCoA (Figure 5D). The results revealed that the diversity of fungal community structure increased following sweating process.

**Figure 6:**
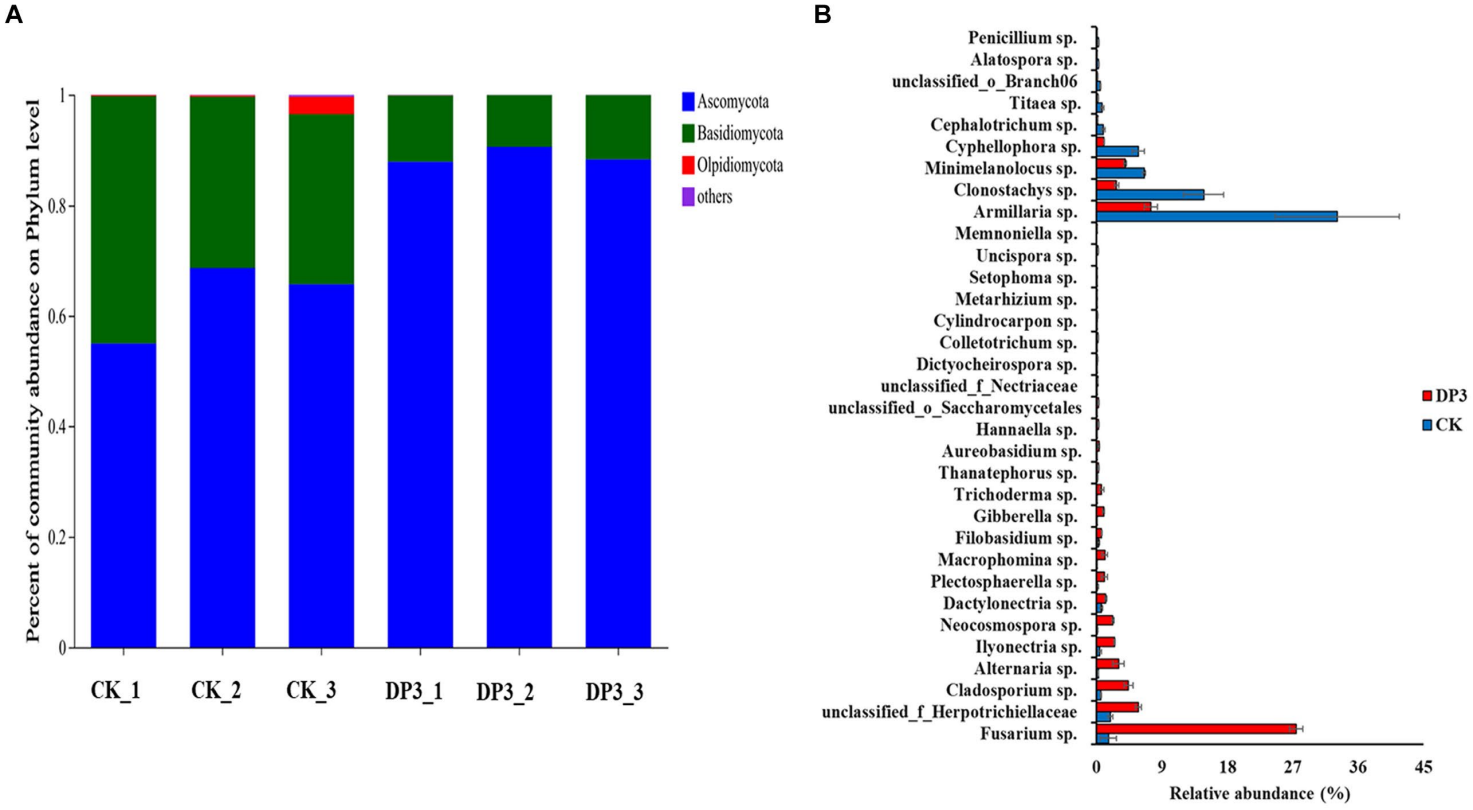
The composition of fungal communities in control and sweated *D. asper.* **(A)** Percentage of community abundance at the phylum level. **(B)** Fungi with significant relative abundance at the genus level.

Furthermore, the composition of microbial communities in the above two samples was analyzed. The OTUs were divided into four main phyla: Ascomycota, Basidiomycota, Olpidiomycota, and others (Figure 6A). There was obvious variation at the phylum level between CK and DP3. The relative abundance of Ascomycota was significantly increased, whereas the relative abundance of Basidiomycota and Olpidiomycota was significantly decreased in DP3. Moreover, the relative abundance of community variation at the genus level had a similar variation to that at the phylum level (Figure 6B). The relative abundance of Ascomycota was increased in DP3, including *Fusarium* sp., *Ilyonectria* sp., *Memnoniella* sp., and *Neocosmospora* sp. Additionally, the relative abundance of *Armillaria* sp., which belong to the Basidiomycota, was decreased in DP3. The results showed that the balance between the Basidiomycota and Ascomycota was disrupted by the sweating process in *D. asper*. The fungi with significant relative abundance variation were associated with color change caused by sweating treatment in *D. asper.* Based on the analysis of the microbial communities in the process of sweating in *M. officinalis*, the relative abundance of *Enterobacter*, *Klebsiella*, *Weissella*, *Bacillus*, and *Candida* would be conducive to improving the quality of *M. officinalis* (Wu et al., 2019). Hence, we speculate that differential metabolites may be involved in the variation of microbial communities.

### Correlation analysis of differential metabolites and microbial communities

**Figure 7:**
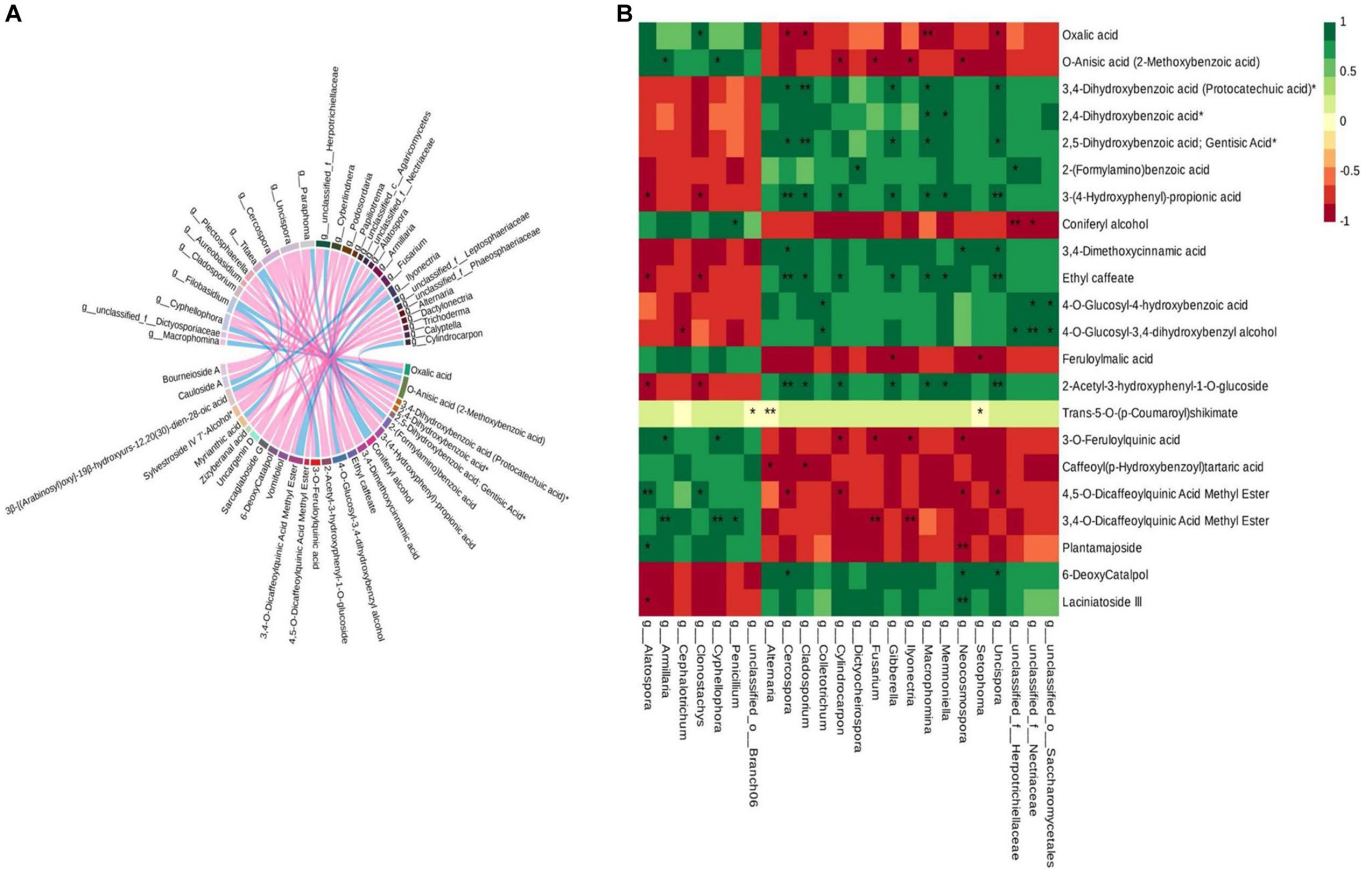
Correlation analysis of the differential metabolites and differential microorganisms in control and sweated *D. asper*. **(A)** Correlation analysis of differential metabolites and differential fungi. **(B)** Correlation analysis of phenolic acids, terpenoids and differential fungi.

Based on the differential metabolite enrichment analysis, the differential phenolic acids and terpenoids may be the key compounds related to the cross-section color change caused by the sweating process. Hence, correlation analysis was conducted between the differential metabolites and the differential microbial communities in control and sweated *D. asper*. The results suggested that the differential fungi of 28 genera correlated with 25 differential metabolites of phenolic acids and terpenoids (Figure 7A). Some fungi that enriched significantly in DP3 showed a significant correlation with these metabolites (Figure 7B). *Neocosmospora* sp. had a significant positive correlation with two iridoid compounds, 6-deoxycatalpol and laciniatoside III, which were upregulated after sweating treatment. Moreover, *Macrophomina* sp. and *Memnoniella* sp. were positively correlated with phenolic acid compounds, including ethyl caffeate, 3-(4-hydroxyphenyl)-propionic acid, 2,4-dihydroxybenzoic acid, and 2-acetyl-3-hydroxyphenyl−1-O-glucoside. Additionally, the results showed that unclassified_o_*Saccharomycetales* and unclassified_f_*Nectriaceae* were positively correlated with 4-O-glucosyl-4-hydroxybenzoic acid and 4-O-glucosyl-3,4-dihydroxybenzyl alcohol. Both *Fusarium* sp. and *Ilyonectria* sp. had a negative correlation with two phenolic acid compounds, 3-O-feruloylquinic acid and 3,4-O-dicaffeoylquinic acid methyl ester, which were downregulated following sweating treatment. Furthermore, some fungi with decreased relative abundance in DP3 showed a significant correlation with metabolites that were downregulated in DP3. The results showed that *Armillaria* sp. and *Cyphellophora* sp. were significantly correlated with O-anisic acid, 3-O-feruloylquinic acid, and 3,4-O-dicaffeoylquinic acid methyl ester. *Penicillium* sp. showed a significant correlation with 3,4-O-dicaffeoylquinic acid methyl ester and coniferyl alcohol. These metabolites were phenolic acids and downregulated in DP3.

It has been reported that *Fusarium* and *Neocosmospora* sp. could colonize pistachio wood and cause vascular discolorations. They were also capable of producing discoloration in stems of clonal rootstocks (Crespo et al., 2019). The discoloration of the stems was similar to the color change of the root that was caused by sweating treatment. Additionally, the study revealed that a gene cluster from *Macrophomina phaseolina* was responsible for the biosynthesis of novel DTAs, macrophasetins (Yu et al., 2022). It has been shown that *M. phaseolina* can produce phomeolic acid in a potato dextrose medium (Singh et al., 2022). *Macrophomina* sp. can produce secondary metabolites, including phenolic acids. In addition, an automatic reconstruction revealed 24 *Penicillium* sp., which have the potential to produce secondary metabolites such as terpenoids and phenylpropanoids (Prigent et al., 2018). Hence, *Macrophomina* sp. and *Penicillium* sp. may have the potential to influence the content of phenolic acids in *D. asper* root with sweating treatment. Therefore, we speculated that 24 fungi interact with 22 differential metabolites to regulate the cross-section color change caused by the sweating processing in *D. asper.*

## Conclusion

In this study, color change was the key phenotype in the sweating process. A total of 667 metabolites in *D. asper* following sweating were identified by widely targeted metabolomics. The content of terpenoids and phenolic acids was involved in green color formation after sweating treatment. In addition, microbial community diversity and richness were increased after sweating treatment. Correlation analysis revealed that the fungi from the Basidiomycota and Ascomycota with the significant relative abundance variation were associated with the content of terpenoids and phenolic acids, which caused the color change following sweating treatment in *D. asper.* This study provides a foundation for analyzing the mechanism in the processing of Chinese medicinal materials.

## Data availability statement

The datasets presented in this study can be found in online repositories. The names of the repository/repositories and accession number(s) can be found below: https://www.ncbi.nlm.nih.gov/sra/,PRJNA1005339.

## Author contributions

HH, JX, and TaoZ conceived and designed the study. HH wrote the manuscript. TmZ and HH performed the experiments. JX, CY, and LL contributed to the data analysis. YY, CX, and CZ revised the manuscript. All authors contributed to the article and agreed to the published version of the manuscript.

## Funding

This study was supported by the National Natural Science Foundation of China (81860675 and 82160725), Guizhou Provincial Science and Technology Projects (Qian Ke He Ji Chu [2020]1Y370, [2019]1026), Research Platform Team Project of the Education Department of Guizhou Province [Qian Jiao Ji [2022]021], and High-Level Innovative Talents of Guizhou Province of China (Qian Ke He Ping Tai Ren Cai [2018] 5638–2).

## Conflict of interest

The authors declare that the research was conducted in the absence of any commercial or financial relationships that could be construed as a potential conflict of interest.

## Publisher’s note

All claims expressed in this article are solely those of the authors and do not necessarily represent those of their affiliated organizations, or those of the publisher, the editors and the reviewers. Any product that may be evaluated in this article, or claim that may be made by its manufacturer, is not guaranteed or endorsed by the publisher.
