# Supplementary material for: Metabolomic and microbiomic insights into color changes during the sweating process in Dipsacus asper
Source: Front Microbiol. 2023 Aug 29;14:1195088. doi: 10.3389/fmicb.2023.1195088 (PMC10499524; doi:10.3389/fmicb.2023.1195088)
Supplement: Supplementary file 2 [file Data_Sheet_1.docx]

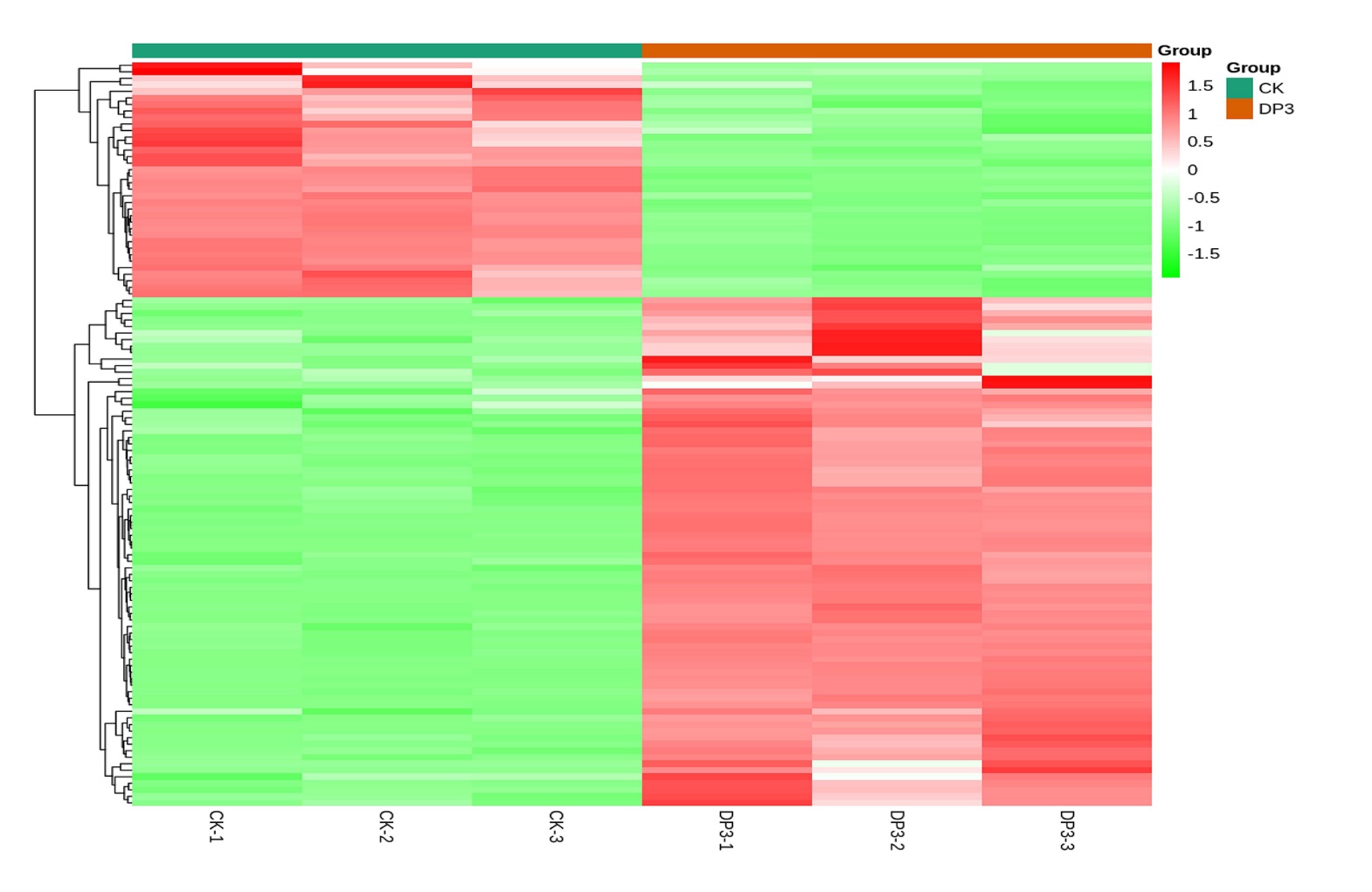


**Figure S1.** Cluster analysis of differential metabolites.


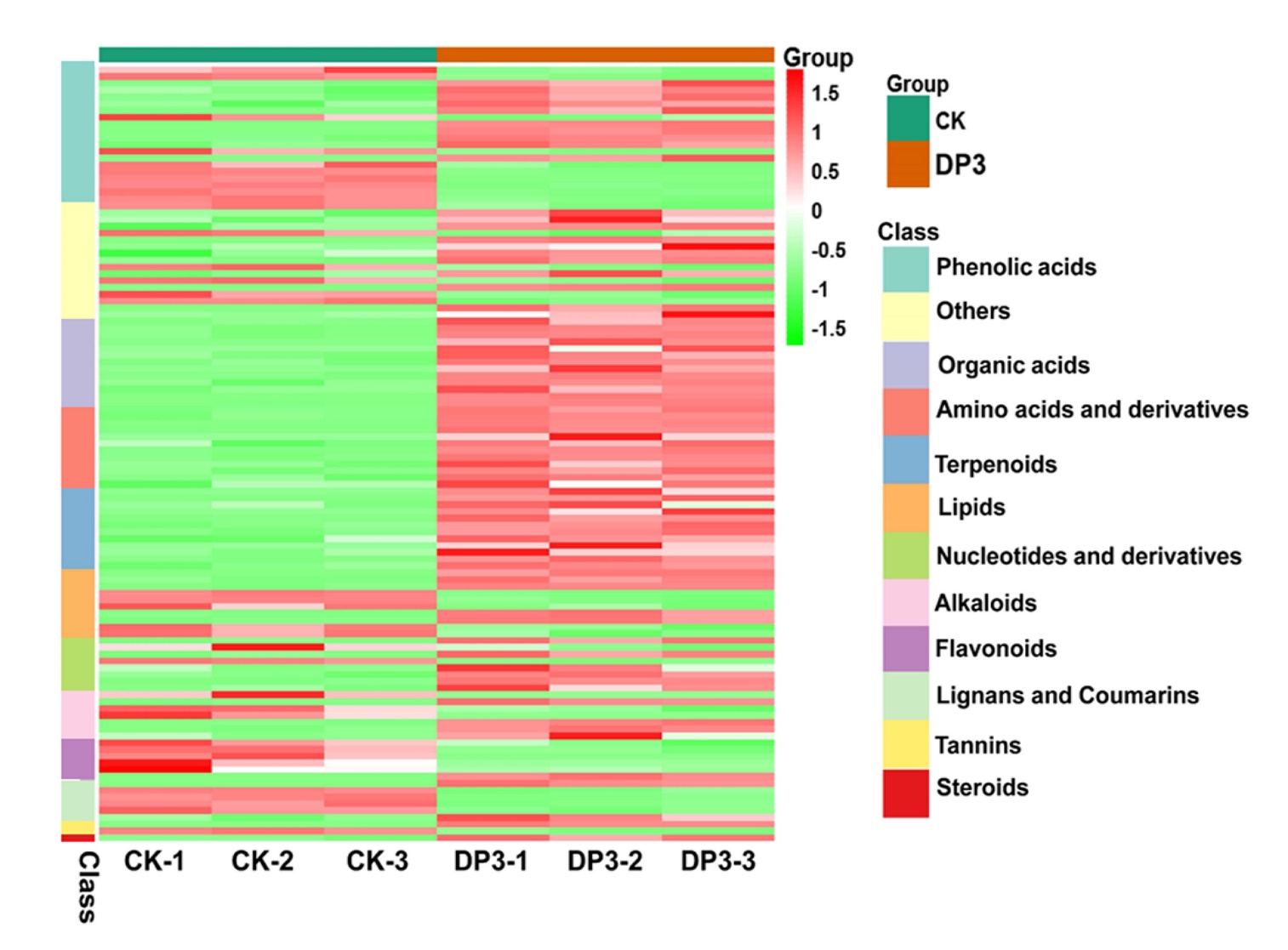


**Figure S2.** Classification of differential metabolites.


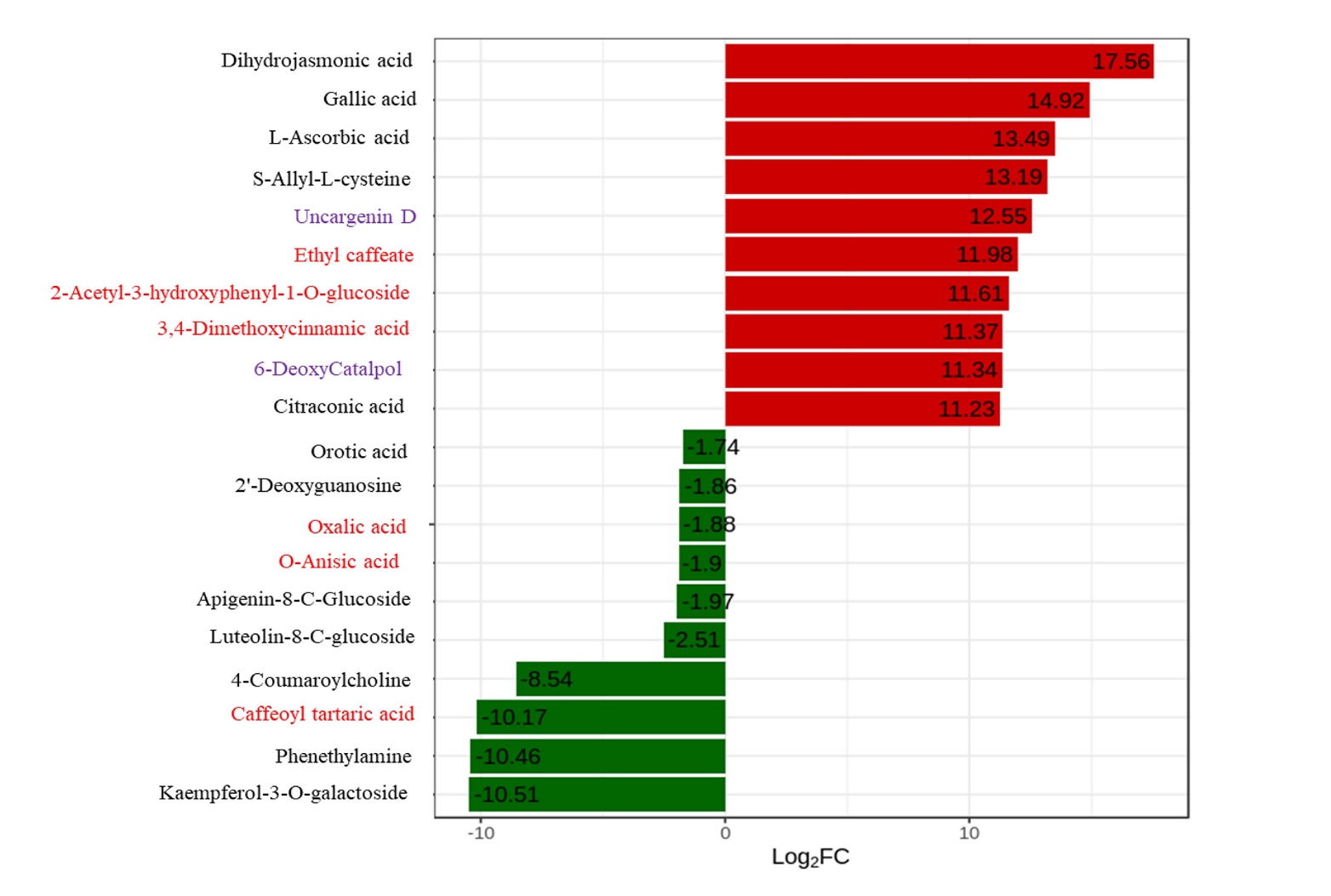


**Figure S3.** Top fold change metabolites. Red represents phenolic acids and purple represents terpenoids.
